# Applying a Systems Perspective to Preventive Health: How Can It Be Useful? Comment on "What Can Policy-Makers Get Out of Systems Thinking? Policy Partners’ Experiences of a Systems-Focused Research Collaboration in Preventive Health"

**DOI:** 10.34172/ijhpm.2020.120

**Published:** 2020-07-13

**Authors:** Matt Egan, Elizabeth McGill

**Affiliations:** ^1^Department of Public Health, Environments and Society, Faculty of Public Health and Policy, London School of Hygiene & Tropical Medicine, London, UK.; ^2^Department of Health Services Research and Policy, Faculty of Public Health and Policy, London School of Hygiene & Tropical Medicine, London, UK.

**Keywords:** Preventive Health, Systems Thinking, Complexity Science, Evidence-Informed Policy

## Abstract

Advocates suggest that a paradigm shift in preventive health towards systems thinking is desirable and may be underway. In a recent study of policy-makers’ opinions, Haynes and colleagues found a mixed response to an Australian initiative that sought to apply systems theories and associated methods to preventive health. Some were enthusiastic about systems, but others were concerned or unconvinced about its usefulness. This commentary responds to such concerns. We argue that a systems perspective can help provide policy-makers with timely evidence to inform decisions about intervention planning and delivery. We also suggest that research applying a systems perspective could provide policy-makers with evidence to support planning and incremental decision-making; make recommendations to support intervention adaptability; consider potential barriers due to incoherent systems, and consider the political consequences of interventions.

In their recent article, Haynes and colleagues provide an extremely interesting assessment of policy-makers’ responses to systems thinking, as promoted by The Australian Prevention Partnership Centre. The Centre is a “collaboration to identify systems, strategies and structures for better decision-making in efforts to prevent lifestyle-related chronic disease in Australia.”^1^ The authors argue that applying and embracing systems thinking represents a paradigm shift away from traditional public health “dominated by acute care and epidemiological models that focus on isolating independent actionable causes.”^1^ Systems thinking is depicted as a collaborative effort that involves “viewing health and healthcare as long-term, evolving, contextually embedded and shaped by interconnected forces at micro, meso and macro levels.”^1^

Paradigm shifts, as originally described by Thomas Kuhn, involve changes to ideas, the kinds of people that produce them, and the wider structures and cultural contexts within which they are embedded.^2^ Shifting paradigms can be a contentious and fractious business. We get a sense of this from the policy-makers Haynes and colleagues interviewed. Some respondents described their excitement and enthusiasm towards both The Centre and its approach to systems. Participants described how they valued the collaborative features of systems thinking. Some were excited by the different world view that systems thinking seems to offer and the opportunities provided by The Centre to engage with experts in this area. In the literature, systems thinking and complexity science are sometimes depicted as distinct but intersecting research traditions.^3^ At times, participants responded differently to these traditions. Some participants appreciated mathematical modelling approaches that stem from the complexity science tradition (sometimes called ‘hard’ system approaches). Some voiced their appreciation of ‘soft’ systems approaches^4^ – which emphasise multiple stakeholder viewpoints and collaborative approaches to applying systems thinking to complex problems.^3,4^

However, Haynes et al also identified dissenting voices. Some participants were suspicious of “evangelical researchers”^1^ and their academic-sounding theories. Some considered system maps to be confusing, and responded to claims that the world is complex by saying “We already know that!”^1^ Some questioned the utility of systems thinking and its capacity to provide evidence that can inform specific decisions to improve population health. Haynes and colleagues argue that such “problem solving”^1^ systems research does exist but that more needs to be done to inform policy-makers of the practical utility of systems thinking.

Stakeholder involvement is a crucial element in many approaches to understanding systems. Systems thinking typically emphasises the different perspectives of a range of stakeholders.^3-5^ Stakeholder perspectives are also drawn upon in the development of maps and models associated with the complexity science tradition.^5-9^ If some policy-makers are unconvinced about the practical utility of systems approaches, this is likely to be a barrier to engagement and adoption. With this in mind, we suggest some ways that systems thinking and complexity science can help inform decisions to improve preventive health policy.

## Producing Findings on Specific Impacts

To help dispel the (misleading) impression that all systems research can offer is a tangled-looking map and a trite-but-true conclusion that the world is very complex, systems approaches need to address the information needs of stakeholders. A central tenant of evidence-informed policy and practice is that evidence should help decision-makers work towards their policy goals.^10,11^ In preventative health policy, these goals are often (but not exclusively) health-related. Researchers can incorporate many interacting elements into their analysis but still tailor their findings to focus on specific elements of a system that are crucial to health decision-makers. These may be health impacts (eg, rates of cardiovascular disease within a population over time^7^) or health behaviours (eg, purchasing of obesogenic foods or beverages^6^), but can also include other impacts relevant to decisions – as identified by the stakeholders involved.^12^

## Modelling Impacts of Unimplemented Policies/Interventions

Preventive health policies and interventions (particularly ‘upstream’ approaches such as investment in the urban environment and infrastructure) are often expensive, can take years to implement, affect large populations and are difficult to undo.^13^ Health impact assessments are already well-established tools for public health decision-makers who want to consider the potential consequences of their decisions. Complexity science provides additional means for modelling potential impacts of planned interventions. These models cannot truly capture the complexities and unpredictability of the real world, but they may be useful for decision-makers to explore through simulation the different policy options they are considering. Different interventions or combinations of interventions can be modelled and compared,^7^ or tested in models designed to simulate different contextual characteristics: for example, simulating the impact of a hypothesised sugar-sweetened beverage intervention in three cities^6^; or the impact of high street tobacco restrictions in different communities.^9^

## Incremental Learning During Implementation

The implementation of preventative health policies and interventions often involves many incremental decisions. Decisions about how or whether to extend the lifespan of an intervention, bring in new partners, modify it for new populations, and whether to scale up or scale down implementation, may need to be taken. Systems approaches provide opportunities for incremental learning to inform decision-making.^13^ This could include further modelling to test new scenarios. It could involve further engagement with different stakeholders to help identify new developments and elicit different views about their consequences.^14^ Systems approaches often emphasise continuous learning cycles to inform adaptations to new or changing contexts. Bringing stakeholders together also provides an opportunity for them to learn from each other, helping to foster mutual understanding and joined-up working.^4,14-16^

## Understanding Processes and Contexts From a Systems Perspective

Understanding processes and contexts, and how they interact to influence impacts, can also aid decision-making. From a systems perspective, impacts are assumed to feedback in ways that affect contextual factors and implementation processes. For instance, an intervention that is considered acceptable and effective in its early stages may consequently attract greater investment, which intensifies the intervention in some way. If an intervention is less well-regarded, funding could be cut or the intervention may be changed. However, these are rather straightforward examples; in a complex world, the story of *what happens* to an intervention and its impacts over time is likely to be more nuanced and unpredictable.^17^

Process evaluations have often been used to provide nuanced assessments of an intervention’s implementation. Typically, they involve qualitative or mixed-methods approaches and often include data collection from both implementers and users. There are some examples of researchers attempting to apply systems thinking to process evaluations^18,19^ but – at least within the field of preventative health policy – this is a relatively under-developed area. Applying systems thinking to process evaluations can involve sampling a wide range of stakeholders. Conceptual tools drawn from systems thinking and complexity science can be applied to the analysis, to help shed light on how an intervention’s implementation interacts with a wider changing system.^13^ Evaluations can also involve a ‘developmental evaluation’ approach, where the research focus can change mid-course in order to examine important emerging developments.^14^

## Unexpected Events

“Events, my dear boy, events” is what the former UK Prime Minister, Harold Macmillan, is famous for saying was most likely to knock governments off course (although the origins of this much-repeated phrase are disputed^20^). For pre-planned evaluations, events suddenly impacting on a system can pose a major problem. Many researchers and policy-makers are currently witnessing the disruptive power of events first-hand as they come to terms with the impact of coronavirus disease 2019 (COVID-19) on their current research area or population of interest. Disruption from less extreme events (eg, new laws, new governments, new technology) is, of course, more common. Unexpected events may originate externally to the system initially envisaged or they may be prompted by emergent behaviours within the system. Developmental evaluations designed to assess system-wide changes and shift focus onto emergent issues are well suited for identifying and considering how stakeholders have responded, or might respond, to such events.^14^

## Coherent and Incoherent Systems

A sporting event used to promote physical activity but prominently sponsored by a fast-food chain can be theorised as being incoherent – impacting on systems that encourage both healthy and unhealthy lifestyles. A community empowerment intervention may appear internally coherent, but if it is implemented while public spending on welfare and local services is cut, there remains a problem of system-level incoherence. Implementers and communities may find themselves swimming against the tide – when even their best efforts are undermined by a wider system that diminishes the impact of their activities. A process evaluation that applies systems thinking could seek to critically examine both the horizontal (eg, community level) and vertical (eg, macro/national level) barriers to impact.^21^ It might help implementers see the value of small improvements in the face of adversity, while building the case for changing vertical structures and policies.

## Political Consequences

Evaluations that focus narrowly on an intervention and its impacts usually have a specific end point: a time when ‘final outcomes’ are measured and impacts over a preceding time period are calculated. However, the intervention and its interactions with the wider system may continue to evolve after the evaluation has ceased – often influenced by some of the issues we have already discussed such as adaptation and emergence.^13^ We would argue that sometimes the most important legacy of an intervention is its impact on political discourse. Stakeholders representing a range of interests may – and do – select and frame research findings into narratives that either support or contest particular health policies and interventions.^22^ Small interventions may gain a new significance as they become incorporated into justifications for or against a particular approach. For example, a local, modestly successful public smoking ban could (hypothetically) be used to support claims that national-level intervention is unnecessary (because it can be addressed locally) or desirable (because effects may be increased if delivered on a larger scale). Even complexity research has at times been reframed by commercial interests (eg, gambling, soft drinks, alcohol) to oppose specific regulatory interventions on the grounds that single interventions are “too simple” to influence complex public health problems.^23^ Widening the scope of process evaluations to consider such framings and their impacts on the wider system would, we argue, move evaluators and their policy partners closer to understanding broader, long-term consequences of an intervention.

## Influencing Rather Than Solving

Haynes and colleagues assume that preventive health policy is a complex policy area, but their article did not explicitly distinguish between complex and complicated systems. Given that some policy-makers found such academic discussions off-putting, this may well have been a sensible omission. A ‘complicated’ system may be made up of many connected parts, but these work together in a predictable way. Complicated problems are characterised as having discreet causes that can be (potentially) solved.^15^ A complex system exhibits behavioural patterns and properties that emerge from, but cannot be reduced to, the individual parts of the system. Complex systems are self-organising and cannot be fully known or controlled. So tackling complex problems should focus on finding ways to influence the system - rather than finding permanent solutions.^16^ As population health problems are widely understood to persist despite repeated efforts to tackle them, this emphasis on influence rather than solution is one that policy-makers can presumably relate to.^24^ Specific policies and interventions may usefully contribute to this influence but the system will continue to adapt, leading to further challenges and opportunities. This further suggests the need for adaptive approaches to influencing the system over the long term as new issues emerge.^4,15^

## Conclusion

As Weiss^10^ might argue, some participants in Hayne and colleagues’ study see how systems thinking can be utilised through an “enlightenment model”: one that emphasises ideas that permeate the policy process over time. They appear willing to accept that systems thinking can encourage an ‘interactive’ approach bringing many different stakeholders together to influence preventive health policy. However, many fail to see evidence of systems thinking leading to research that directly informs decisions and solves specific problems. Communicating the potential of systems thinking to inform decision-making is therefore important. Demonstrating that potential is more important still.

## Ethical issues

Not applicable.

## Competing interests

ME and EM report grants from the National Institute for Health Research (NIHR) School for Public Health Research.

## Authors’ contributions

ME and EM both contributed to conception and design, analysis and interpretation, drafting and critical revision of the manuscript.

## Disclaimer

The views expressed are those of the author(s) and not necessarily those of the NIHR or the Department of Health and Social Care.

## Funding

This study was supported by the NIHR School for Public Health Research (SPHR), Grant Reference Number PD-SPH-2015.

## Authors’ affiliations

^1^Department of Public Health, Environments and Society, Faculty of Public Health and Policy, London School of Hygiene & Tropical Medicine, London, UK. ^2^Department of Health Services Research and Policy, Faculty of Public Health and Policy, London School of Hygiene & Tropical Medicine, London, UK.
